# Diversity of *ATM* gene variants: a population-based genome data analysis for precision medicine

**DOI:** 10.1186/s40246-019-0234-2

**Published:** 2019-08-23

**Authors:** Hisanori Fukunaga, Yasuyuki Taki, Kevin M. Prise

**Affiliations:** 1 Centre for Cancer Research and Cell Biology, Queen’s University Belfast, 97 Lisburn Road, Belfast, BT9 7AE UK; 2 Shonan Kamakura General Hospital, 1370-1 Okamoto, Kamakura, Kanagawa 247-8533 Japan; 3 Institute of Development, Aging and Cancer, Tohoku University, 4-1 Seiryo-machi, Aoba-ku, Sendai, 980-8573 Japan; 4 Tohoku Medical Megabank Organization, Tohoku University, 2-1 Seiryo-machi, Aoba-ku, Sendai, 980-8575 Japan

**Keywords:** Ataxia-telangiectasia mutated, Population-based biobank, Heterozygotes, Precision medicine, Whole-genome reference panel

## Abstract

**Background:**

Ataxia-telangiectasia (AT) is a rare autosomal recessive disorder that causes deficiency or dysfunction of the ataxia-telangiectasia mutated (ATM) protein. Not only AT patients, but also certain *ATM* heterozygous mutation carriers show a significantly reduced life expectancy due to cancer and ischemic heart disease; in particular, female carriers having particular alleles have an increased risk of breast cancer. The frequency of such risk heterozygotes at a population level remains to be fully determined, and evidence-based preventive medical guidelines have not yet been established.

**Methods:**

Using the 3.5KJPNv2 allele frequency panel of Japanese Multi Omics Reference Panel v201902, which shows single-nucleotide variant (SNV) and insertion/deletion (INDEL) allele frequencies from 3552 Japanese healthy individuals, we investigated the diversity of *ATM* gene variants.

**Results:**

We detected 2845 (2370 SNV and 475 INDEL) variants in the *ATM* gene, including 1338 (1160 SNV and 178 INDEL) novel variants. Also, we found a stop-gained SNV (NC_000008.11:g.108115650G > A (p.Trp266*)) and a disruptive-inframe-deletion (NC_000008.11:g. 108181014AAGAAAAGTATGGATGATCAAG/A (p.Ala1945_Phe1952delinsVal) and two frameshift INDELs (NC_000008.11:g.108119714CAA/C (p.Glu376fs) and NC_000008.11:g.108203577CTTATA/C (p.Ile2629fs)), which would be novel variants predicted to lead to loss of ATM functionality.

**Conclusion:**

The combination of population-based biobanking and human genomics provided a novel insight of diversity of *ATM* gene variants at a population level. For the advancement of precision medicine, such approach will be useful to predict novel pathogenic/likely pathogenic variants in the *ATM* gene and to establish preventive medical guidelines for certain *ATM* heterozygotes pertaining to their risk of particular diseases.

## Introduction

Ataxia-telangiectasia (AT) is a rare autosomal recessive disorder clinically characterized by cerebellar degeneration, immune-deficiency, radiation hypersensitivity, and cancer predisposition. Also, metabolic disorders implicated in cardiovascular and liver diseases are frequently observed in adolescent AT patients [1]. The worldwide prevalence of AT is estimated to be between 1 in 40,000 and 1 in 100,000 live births [2].

The *ataxia-telangiectasia mutated (ATM)* gene was first reported in 1995 as the causative gene responsible for AT [3]. Most AT patients lack functional ATM protein due to missense or non-sense mutations in the *ATM* gene, which result in truncated or unstable ATM variants [4]. The *ATM* gene is located on chromosome 11q22-q23, spanning approximately 150 kb of genomic DNA, and encodes a protein of 3056 amino acids which is a phosphoinositidyl 3-kinase (PI3K)-family kinase [5]. ATM forms dimers or oligomers under non-stress conditions, and DNA damage induces intermolecular autophosphorylation of Ser1981 that causes dimer dissociation and initiates intracellular ATM kinase activity [6]. In addition to Ser1981, other autophosphorylation sites, Ser367, Ser1893, and Ser2996 are physiologically important parts of the DNA damage response [7, 8]. Also, ATM phosphorylates several proteins involved in cell cycle checkpoint control, apoptosis, and DNA repair, including p53, Chk2, BRCA1, RPAp34, H2AX, SMC1, FANCD2, Rad17, Artemis, and Nbs1 [9].

Not only AT patients, but also certain *ATM* heterozygous mutation carriers have a reduced life expectancy. In 1987, Swift and co-workers reported that for heterozygotes responsible for AT, the relative risk of cancer was estimated to be 2.3 for men and 3.1 for women [10]. Furthermore, according to a systematic review in 2016, siblings of AT patients who are heterozygous carriers of pathogenic *ATM* gene mutations have a significantly increased risk of cancer and ischemic heart disease (relative risk (RR) 1.7, 95% confidential interval (CI) 1.2–2.4), and female heterozygotes having particular alleles have an increased risk of breast cancer (RRwomen 3.0, 95% CI 2.1–4.5) [11]; thus, certain *ATM* heterozygous mutation carriers should be made aware of lifestyle factors that contribute to the development of such diseases. However, the accurate frequency of heterozygous carriers of pathogenic mutations in the *ATM* gene at a population level, to date, remains to be determined, and evidence-based preventive medical guidelines have not yet been established [12]. From the point of view of precision medicine, appropriate approaches are immediately needed for *ATM* heterozygotes pertaining to their risk of particular diseases.

## Methods

To address the issue, we used the 3.5KJPNv2 allele frequency panel [13–16], which is a part of Japanese Multi Omics Reference Panel version 201902 (Feb 2019, https://jmorp.megabank.tohoku.ac.jp/201902/) released from the Tohoku University’s Tohoku Medical Megabank Organization (ToMMo) [17]. This population-based whole-genome reference panel shows single-nucleotide variant (SNV) and insertion/deletion (INDEL) allele frequencies from 3552 Japanese healthy individuals. jMorp was originally published in 2018, as a multi-omics database of metabolites and proteins in plasma obtained from volunteers in ToMMo [17]. From jMorp release 201806 (Jun 2018, https://jmorp.megabank.tohoku.ac.jp/201806/), genomic variant data have been added, and the version 201,902 (Feb 2019, https://jmorp.megabank.tohoku.ac.jp/201902/) is where allele frequencies of all the genomic variants can be examined through the web interface.

Although they previously used an original re-sequencing workflow for the 1KJPN5 [13], 2KJPN, and 3.5KJPNv1, for building the 3.5KJPNv2, ToMMo decided to use a more common pipeline including the 1000 Genomes Project [18] and gnomAD [19] algorithms to reduce technical biases and to allow comparisons to other populations. ToMMo customized three steps in the Genome Analysis Toolkit (GATK) Best Practices workflow, which are widely used in large-scale sequencing projects and recommend post-alignment processing before variant calling [20]: (1) the choice of the reference genome, (2) the use of base quality score recalibration (BQSR), and (3) the joint genotyping step. Although the GATK Best Practices workflow recommends that the BQSR step be carried out after the mapping, ToMMo did not do so, but checked concordance among two kinds of genotyping results: (i) genotyping results obtained after the incorporation of BQSR and (ii) results obtained without BQSR. Thus, the allele frequency panel, 3.5KJPNv2 of jMorp version 201902, can be easily used to compare the allele frequencies of different populations with the web interface.

The 3.5KJPNv2 is available at jMorp website, and the raw data in Variant Call Format (VCF) format was also registered at the NBDC Human Database (https://humandbs.biosciencedbc.jp/en/) with accession code hum0015.v3 by the National Bioscience Database Center (NBDC) of the Japan Science and Technology Agency to ensure accessibility, preservation, and stability of the 3.5KJPNv2 datasets [16]. Individual’s sequence data and genotyping results from which allele frequency dataset is constructed and validated are available upon request after approval of the Ethical Committee and the Materials and Information Distribution Review Committee of ToMMo.

In the present study, we accessed the raw data of the 3.5KJPNv2 allele frequency panel and investigated the allele frequencies of *ATM* gene variations, with the assumption of the Hardy-Weinberg equilibrium. Also, we investigated novel variants, which are predicted to lead to loss of ATM functionality based on their protein structures.

## Results and discussion

**Fig. 1:**
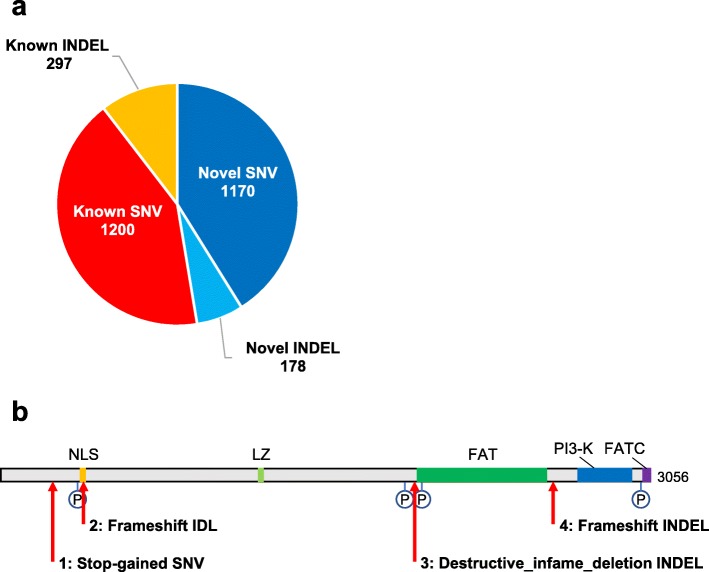
Novel variants predicted to lead to loss of ATM functionality. **a** A total of 2845 (2370 SNV and 475 INDEL) variants in the *ATM* gene has confirmed, including 1338 (1160 SNV and 178 INDEL) novel variants which have not yet been assigned reference SNP ID numbers. **b** ATM is a protein of 3056 amino acids. The phosphorylation sites (P) indicate the positions of serine residues, Ser367, Ser1893, Ser1981, and Ser2996 [6–8]. The NLS (~aa 385 to 388), the LZ (~aa 1216 to 1241), the FAT (~aa 1960 to 2566), the PI3-K (~aa 2712 to 2962), and the FATC ( ~aa 3024 to 3056) domains are shown in orange, light green, green, blue, and violet respectively [9]. Novel variants predicted to lead to loss of ATM functionality are indicated using red arrows: (1) stop-gained SNV, NC_000008.11:g.108115650G > A (p.Trp266*), (2) frameshift INDEL, NC_000008.11:g.108119714CAA/C (p.Glu376fs), (3) disruptive-inframe-deletion INDEL, NC_000008.11:g.108181014AAGAAAAGTATGGATGATCAAG/A (p.Ala1945_Phe1952delinsVal), (4) frameshift INDEL, NC_000008.11:g.108203577CTTATA/C (p.Ile2629fs)

As shown in Fig. 1a, with the 3.5KJPNv2 allele frequency panel, we searched for the allele frequencies of *ATM* gene variations and found 2845 (2370 SNV and 475 INDEL) variants in the given population, including 1338 (1160 SNV and 178 INDEL) novel variants, which have not yet been assigned reference single-nucleotide polymorphism (SNP) ID numbers [21]. This result demonstrates that, with the assumption of the Hardy-Weinberg equilibrium, a large number of healthy individuals have novel heterozygous variants in the *ATM* gene, indicating that the *ATM* genetic diversity is greater than expected.

We found a novel stop-gained SNV in the *ATM* gene, NC_000008.11:g.108115650G > A (p.Trp266*). As shown in Fig. 1b, this SNV removes all the main identified domains including the nuclear localization signal (NLS; ~aa 385 to 388), the leucine zipper (LZ; ~aa 1216 to 1241), the FRAP/ATM/TRRAP (FAT; ~aa 1960 to 2566), the kinase (PI3-K; ~aa 2712 to 2962), and the FAT c-terminal (FATC; ~aa 3024 to 3056) domains, which are physiologically important for the activation and regulation of ATM kinase activity [9]. Also, this removes autophosphorylation sites, Ser367, Ser1893, Ser1981, and Ser2996, which are significantly related to ATM protein functionality. Taken together, the stop-gained SNV in the *ATM* gene is predicted to be a novel loss-of-function variant.

Next, we detected a novel disruptive_inframe_deletion, NC_000008.11:g.108181014AAGAAAAGTATGGATGATCAAG/A (p.Ala1945_Phe1952delinsVal), and two frameshift INDELs, NC_000008.11:g.108119714CAA/C (p.Glu376fs) and NC_000008.11:g.108203577CTTATA/C (p.Ile2629fs). Because of the dramatic changes of amino acid sequence and three-dimensional structure, all the INDELs are predicted to lead to loss of ATM functionality. Even NC_000008.11:g.108203577CTTATA/C, although maintaining more than 2500 normal amino acid sequence from the N-terminal of ATM, removes normal C-terminal PI3-K domain and autophosphorylation site Ser2996, which are essential for ATM signaling in human cells.

To our knowledge, there is no previous study relating to the clinical risk of these four variants. However, according to the possible ATM functionality based on their protein structures, certain *ATM* heterozygous mutation carriers having these variants are likely to have a reduced life expectancy. From the point of view of precision medicine, personalized preventive medical strategies would be immediately needed.

## Conclusion

In this study, we showed the diversity of *ATM* gene variants at a population level and found four novel variants which are predicted to lead to loss of ATM functionality. Further advancements in the combination of population-based biobanking and human genomics are expected to further uncover the genetic basis of AT patients and certain *ATM* heterozygotes who have a reduced life expectancy. Also, for the advancement of precision medicine, such approach will be useful to establish an evidence-based guideline not only for AT patients but also for risk *ATM* heterozygotes seeking preventive medical strategies.

## Data Availability

Please contact ToMMo (https://www.megabank.tohoku.ac.jp/english/) for additional information.

## Abbreviations

AT: Ataxia-telangiectasia
ATM: Ataxia-telangiectasia mutated
BQSR: Base quality score recalibration
CI: Confidential interval
DNA: Deoxyribonucleic acid
FAT: FRAP/ATM/TRRAP
FATC: FAT c-terminal
GATK: Genome Analysis Toolkit
Indel: Insertion/deletion
jMorp: Japanese Multi Omics Reference Panel
LZ: Leucine zipper
NBDC: National Bioscience Database Center
NLS: Nuclear localization signal
PI3K: Phosphoinositide 3-kinase
RR: Relative risk
SNP: Single-nucleotide polymorphism
SNV: Single-nucleotide variant
ToMMo: Tohoku Medical Megabank Organization
